# Dietary charcoal supplementation modulates hematological, biochemical, immunological, antioxidant, and intestinal parameters in ducks

**DOI:** 10.5455/javar.2025.l934

**Published:** 2025-08-18

**Authors:** Mohamed Hosny, Mohamed F.A. Farghly, Mostafa G. Abdelfattah, Mohamed E. Abd El-Hack, Elwy A. Ashour, Ahmed K. Aldhalmi, Ayman A. Swelum, Vincenzo Tufarelli, Mohamed S. El-kholy, Alessandra Tateo

**Affiliations:** 1 Department of Animal Production, Faculty of Agriculture, Al–Azhar University, Assiut, Egypt; 2 Department of Poultry Production, Faculty of Agriculture, Assiut University, Assiut, Egypt; 3 Department of Poultry, Faculty of Agriculture, Zagazig University, Zagazig, Egypt; 4 Department of Industrial Pharmacy, College of Pharmaceutical Sciences and Drug Manufacturing, Misr University for Science and Technology (MUST), Giza, Egypt; 5 College of Pharmacy, Al–Mustaqbal University, Hillah, Iraq; 6 Department of Animal Production, College of Food and Agriculture Sciences, King Saud University, Riyadh, Saudi Arabia; 7 Department of Precision and Regenerative Medicine and Jonian Area, Section of Veterinary Science and Animal Production, University of Bari Aldo Moro, Bari, Italy

**Keywords:** Antioxidant status, charcoal, duck, health, immunity

## Abstract

**Objective:**

This study aimed to investigate the impact of dietary charcoal supplementation on hematological and biochemical indices, immune function, antioxidant status, and intestinal histomorphology in ducks.

**Materials and Methods:**

A total of 144 mule ducks, aged 4 weeks, were randomly allocated into 6 experimental groups. Birds were reared under uniform conditions in floor pens and provided with a basal diet (3,000 kcal/kg ME and 20% CP) supplemented with 0.0%, 0.5%, 1.0%, 1.5%, 2.0%, or 2.5% charcoal.

**Results:**

Dietary inclusion of charcoal at 1.0%, 1.5%, and 2.0% significantly reduced serum creatinine (*p *= 0.049) and urea concentrations (*p *= 0.036), suggesting enhanced renal function. Additionally, ducks receiving 1.5% and 2.0% charcoal exhibited significantly lower plasma corticosterone levels (*p* = 0.045) and elevated blood glucose concentrations (*p* = 0.042) compared to the control group. No significant differences (*p* > 0.05) were observed in other serum biochemical markers (total protein, albumin, globulin, albumin-to-globulin ratio, cholesterol, aspartate aminotransferase, and alanine aminotransferase) or hematological parameters. Antioxidant capacity was significantly enhanced (*p* ≤ 0.05) in ducks fed 1.5% charcoal, except for malondialdehyde levels, which remained unaffected (*p* = 0.943). Serum immunoglobulin A concentrations and relative spleen weights were significantly higher in the 1.5% and 2.0% charcoal groups (*p* = 0.0125 and *p *= 0.0207, respectively), while IgG and IgM levels did not differ among treatments (*p* > 0.05). Moreover, charcoal supplementation at 1.5%, 2.0%, and 2.5% positively influenced the villus architecture of both the duodenum and cecum (*p* < 0.05).

**Conclusion:**

Dietary supplementation with up to 1.5% charcoal appears to confer physiological benefits in ducks by supporting renal function, enhancing immune and antioxidant responses, and improving intestinal morphology.

## Introduction

Charcoal, primarily composed of carbon (approximately 70%–90%), also contains trace minerals such as manganese, potassium, calcium, and zinc [1]. Its highly porous microstructure, with a broad distribution of pore sizes and shapes, allows it to adsorb a variety of chemical and biological molecules [2]. Due to its capacity to bind substances non-specifically within the gastrointestinal tract, charcoal has historically been utilized as an oral detoxifying agent to minimize the systemic absorption of toxins [3].

This adsorptive property contributes to several physiological effects, including the stabilization of intestinal membranes and the reduction of surface tension in the gut. Charcoal facilitates the elimination of harmful gases, toxins, and metabolic by-products, which may, in turn, enhance the efficiency of nutrient absorption through the intestinal lining [4].

Furthermore, its ability to bind antinutritional compounds and toxic metabolites suggests a role as a functional additive that can improve gut health and reduce digestive stress [4]. It has also been shown to act as a non-selective adsorbent of microbial pathogens [5] and to bind residual hazardous substances in animal feed, potentially improving feed quality and safety [6]. These features highlight its promise as a natural and economical alternative to synthetic antibiotics in livestock nutrition.

Previous research has documented the positive effects of dietary charcoal supplementation in a variety of species. In broiler chickens, it has been associated with enhanced growth performance and feed conversion [7], improved immune competence and intestinal microbial balance [8], and better blood and antioxidant profiles in fish species such as Nile tilapia [9]. Positive changes in intestinal architecture have been reported in laying hens [10], and reductions in microbial contamination have been observed in duck meat [11].

Despite these findings, evidence regarding the application of charcoal in duck nutrition remains scarce. Therefore, the present study was conducted to assess the effects of dietary charcoal supplementation on hematological and biochemical parameters, immune response, antioxidant capacity, and gut morphology in ducks.

## Materials and Methods

### Ethical approval

The study was reviewed and approved by the Institutional Ethics Committee of the Department of Poultry Production, Faculty of Agriculture, Assiut University, Assiut, Egypt (IACUC).

### Experimental design and animal husbandry

A total of 144 Mule ducks, aged 4 weeks, were selected to investigate the effects of dietary charcoal supplementation on hematological and biochemical indices, immune response, antioxidant status, and intestinal morphology. The birds were randomly assigned to six experimental groups (G1–G6), each comprising 24 ducks, further divided into 3 replicates of 8 individuals. All birds were housed in uniform floor pens under standardized management conditions. The dietary treatments consisted of a standard basal diet supplemented with increasing levels of charcoal: 0% (control), 0.5%, 1.0%, 1.5%, 2.0%, and 2.5% for G1–G6, respectively. The charcoal used was produced through high-temperature exposure to an oxidizing gas mixture, enhancing its porosity and surface area [12]. Its proximate composition included 99.02% dry matter, 1.98% crude protein, 11.22% crude fiber, 0.00% ether extract, and 2.09% ash. Throughout the experimental period, feed and water were provided *ad libitum*. The basal diet was formulated to meet or exceed the nutrient requirements for ducks as outlined by the National Research Council [13], delivering 3,000 kcal/kg of metabolizable energy and 20% crude protein up to 16 weeks of age. The lighting schedule followed a 16-h light and 8-h dark cycle (16L:8D) with an intensity maintained between 10 and 20 lux/m^2^ [11].

### Blood sampling and analysis

After the experiment (week 16), blood samples were collected during the slaughtering process using sterile, heparinized tubes. Whole blood was analyzed to determine red blood cell (RBC, 10^6^/µl) and white blood cell (WBC, 10^3^/µl) counts [14]. Hemoglobin concentration (Hb, gm/dl) was measured [15], while packed cell volume (PCV, %) was assessed [16]. For serum collection, blood was transferred into tubes without an anticoagulant and centrifuged at 4,000 rpm for 15 min. The harvested serum was stored at −20°C until further biochemical analyses.

Serum total protein levels (gm/dl) were quantified utilizing the Biuret colorimetric method [17], whereas serum albumin (gm/dl) was determined via the colorimetric approach [18]. Globulin concentration (gm/dl) was calculated by subtracting albumin from total protein, and the albumin-to-globulin (A:G) ratio was subsequently derived. Total cholesterol (mg/dl) was measured following standard procedures [19]. Activities of serum aspartate aminotransferase (AST, IU/ml) and alanine aminotransferase (ALT, IU/ml) were evaluated [20]. Serum creatinine (mg/dl) and urea (mg/dl) concentrations were assessed, with urea quantified using the Urease–Berthelot assay [21,22]. Serum corticosterone levels (ng/ml) were determined via a commercial ELISA kit in accordance with the manufacturer's instructions. Glucose concentration (mg/dl) was measured [23].

Markers of oxidative status were analyzed using established protocols: total antioxidant capacity (T-AOC, nmol/ml) [24], malondialdehyde (MDA, nmol/ml) [25], glutathione peroxidase activity (GSH-Px, U/ml) [26], superoxide dismutase (SOD, U/ml) [27], and catalase (CAT, U/ml) [28]. Serum immunoglobulin concentrations (IgG, IgM, and IgA, mg/dl) were measured using commercial biodiagnostic kits [29].

### Relative weight of lymphoid organs

Following slaughter, lymphoid organs—including the spleen, thymic lobes, and bursa of Fabricius—were carefully excised from each duck. After the removal of any adhering connective tissue, the organs were weighed individually. Relative organ weights were then calculated and expressed as a percentage of the bird’s live body weight.

### Intestinal morphology

From each slaughtered duck (three birds per treatment group), approximately 1 cm segments were collected from the midpoint of each section of the small intestine (duodenum, jejunum, and ileum) as well as the cecum. Samples were rinsed with distilled water and immediately fixed in 10% neutral buffered formalin. After fixation, tissues were processed through paraffin embedding, and 5 mm sections were prepared. Using an RM2245 rotary microtome, sections were cut at a thickness of 6 µm and mounted on glass slides. The mounted sections were stained with hematoxylin and eosin and then dried at 37°C for 12 h, followed by a 2-min xylene treatment for clearing, following the protocol of Suvarna et al. [30]. Histological evaluation was performed using a light microscope (Olympus, Tokyo, Japan) to measure villus height (V, µm) and crypt depth (C, µm) based on the method [31]. Subsequently, the villus height to crypt depth ratio (V:C) was calculated.

### Statistical analysis

The study employed a completely randomized design. Data obtained for all measured parameters were analyzed using a one-way analysis of variance to identify statistically significant differences among treatment groups at the 0.05 significance level. Where significant differences were observed, Duncan’s multiple range test was applied for pairwise comparisons of means [32]. All statistical analyses were performed using SAS software version 9.2 [33].

## Results and Discussion

### Blood biochemical and hematological parameters

Blood hematochemical profiles serve as crucial indicators for assessing the physiological and health status of animals [9]. In the present study, except for creatinine, urea, glucose, and corticosterone, no significant alterations were detected among the experimental groups for the majority of blood parameters evaluated (Table 1). Dietary inclusion of charcoal at concentrations of 1%, 1.5%, and 2% resulted in a significant reduction in serum creatinine (*p *= 0.049) and urea levels (*p* = 0.036) relative to the control group. Conversely, supplementation with 0.5% and 2.5% charcoal did not produce significant differences in these parameters compared to controls.

The observed decrease in creatinine and urea suggests an enhancement of renal function, potentially attributable to the adsorptive properties of charcoal toward toxins [4] and pathogenic bacteria [5]. This phenomenon, often referred to as "intestinal dialysis," involves the binding of urea and other metabolic waste products to charcoal within the gut, facilitating their elimination via feces [12]. Supporting this, El-Kafoury et al. [34] demonstrated that activated charcoal can slow chronic kidney disease progression in albino rats by limiting the intestinal absorption of bacterial toxins into systemic circulation.

Regarding stress and energy metabolism markers, ducks receiving 1.5% (G3) and 2% (G4) charcoal exhibited significantly lower serum corticosterone levels (*p* = 0.045) alongside elevated glucose concentrations (*p* = 0.042) compared to the control group (G1). Other charcoal treatment groups (0.5%, 1%, and 2.5%) showed no significant differences in corticosterone or glucose relative to controls. The increased glucose levels in certain groups may be linked to an enhanced absorptive surface area within the intestine following charcoal supplementation. Corticosterone, a glucocorticoid hormone secreted by the adrenal cortex in response to stress, was reduced in the 1.5% and 2% charcoal groups, potentially reflecting charcoal’s capacity to mitigate stress-related oxidative damage, as corroborated by antioxidant data (Table 3).

No significant differences (*p* > 0.05) were observed among all groups in hematological indices such as RBCs, WBCs, Hb concentration, and PCV (Table 2). The highest—albeit not statistically significant—RBC and Hb values were found in ducks fed the 1.5% charcoal diet.

These findings align with those reported by Ruben et al. [35], who documented reduced serum creatinine levels in broilers fed 0.2% charcoal without significant changes in total protein, globulin, AST, ALT, urea, or hematological parameters. Similarly, Enyenihi et al. [36] found no significant effects of varying charcoal levels (0%–8%) on blood RBCs, WBCs, Hb, or PCV in broiler chickens. Chu et al. [37] also observed comparable corticosterone levels in fattening pigs supplemented with 0.3% bamboo charcoal.

### Antioxidant capacity

Results presented in Table 3 demonstrate that dietary charcoal supplementation significantly influenced serum antioxidant parameters, including (T-AOC, *p* = 0.014), (GSH-Px, *p* = 0.003), (SOD, *p* = 0.002), and (CAT, *p* = 0.045), whereas MDA levels remained unaffected (*p* = 0.943). T-AOC values were elevated in ducks receiving 1% (G2) and 1.5% (G3) charcoal compared to the control (0% charcoal) and the 2.5% charcoal group (G6). Other treatment groups supplemented with 0.5%, 2%, and 2.5% charcoal showed T-AOC levels comparable to the control.

**Table 1.** Effect of graded levels of charcoal on some blood biochemistry of ducks.

*p*-value	SEM	Charcoal levels						Traits
		G6 2.5%	G5 2.0%	G4 1.5%	G3 1.0%	G2 0.5%	G1 0%	
0.1256	0.39	4.1	4.11	3.96	4.04	3.95	3.97	Total proteins (gm/dl)
0.7952	0.25	2.45	2.39	2.35	2.41	2.39	2.46	Albumin (gm/dl)
0.2474	0.19	1.65	1.72	1.61	1.63	1.56	1.51	Globulin (gm/dl)
0.1525	0.21	1.48	1.39	1.46	1.48	1.53	1.63	A:G ratio
0.2151	11.21	168.8	158.5	155.2	169.4	176.7	174.2	Cholesterol (mg/dl)
0.4194	4.92	36.37	35.69	35.34	40.11	37.52	38.84	AST (IU/ml)
0.5026	2.31	11.12	11.06	10.54	12.41	13.69	14.60	ALT (IU/ml)
0.0492	0.24	2.49^ab^	1.45^c^	1.48^c^	2.04^b^	2.54^a^	2.63^a^	Creatinine (mg/dl)
0.0356	1.12	7.59^ab^	7.04^b^	7.05^b^	7.09^b^	7.79^a^	7.63^ab^	Urea (mg/dl)
0.0452	5.32	37.64^ab^	24.45^b^	25.28^b^	39.24^ab^	39.48^ab^	42.96^a^	Corticosterone (ng/ml)
0.0423	1.15	17.78^ab^	18.91^a^	18.75^a^	17.96^ab^	15.11^b^	14.25^b^	Glucose (mg/dl)

^a, b, c,^ means within row, followed by different superscripts, are significantly different (*p* < 0.05). A:G ratio = Albumin/Globulin ratio; AST = Aspartate aminotransferase; ALT = Alanine transaminase.

**Table 2.** Effect of graded levels of charcoal on some blood hematology of ducks.

*p*-value	SEM	Charcoal levels						Traits
		G6 2.5%	G5 2.0%	G4 1.5%	G3 1.0%	G2 0.5%	G1 0%	
0.0541	0.258	2.41^ab^	2.48^ab^	3.80^a^	3.66^a^	2.64^b^	2.72^b^	RBCs (10^6^/ul)
0.6458	7.962	77.64	74.42	75.35	79.64	78.45	72.84	WBCs (10^3^/ul)
0.4162	0.605	8.60	7.82	8.83	8.76	7.92	8.60	Hb (gm/dl)
0.5264	0.115	35.8	36.3	34.8	35.8	34.6	36.5	PCV (%)

^a, b ^means within row, followed by different superscripts, are significantly different (*p* ≤ 0.05). RBCs = Red blood cells; WBCs = White blood cells; Hb = Hemoglobin value; PCV = Packed cell volume.

**Table 3.** Effect of graded levels of charcoal on the antioxidant capacity of ducks.

*p*-value	SEM	Charcoal levels						Traits
		G6 2.5%	G5 2.0%	G4 1.5%	G3 1.0%	G2 0.5%	G1 0%	
0.0136	0.29	3.14^b^	3.62^ab^	3.99^a^	4.15^a^	3.56^ab^	3.09^b^	T-AOC, (nmol/ml)
0.9426	1.46	16.71	16.07	15.79	15.88	17.01	16.61	MDA, (nmol/ml)
0.0034	12.51	250.1^b^	238.4^b^	294.8^a^	261.0^ab^	207.4^c^	240.6^b^	GSH-Px, (U/ml)
0.0016	10.11	160.0^b^	163.5^b^	186.0^a^	172.0^ab^	159.2^b^	130.0^c^	SOD, (U/ml)
0.0452	1.12	8.59^b^	11.26^a^	11.31^a^	10.42^ab^	8.21^b^	8.60^b^	CAT, (U/ml)

^a, b, c,^ means within row, followed by different superscripts, are significantly different (*p* ≤ 0.05). T-AOC = Total Antioxidant Capacity; MDA = malondialdehyde, GSH-Px = Glutathione peroxidase; SOD = Superoxide dismutase; CAT = Catalase.

GSH-Px activity was significantly higher in the 1.5% charcoal group (G4) relative to controls. No significant differences were observed in GSH-Px activity for groups receiving 1%, 2%, and 2.5% charcoal; meanwhile, the 0.5% charcoal group recorded the lowest enzyme activity among all treatments, including the control. SOD activity increased markedly across all charcoal-supplemented groups, with the 1.5% charcoal group displaying the highest levels. CAT activity was elevated in birds fed 1.5% (G4) and 2% (G5) charcoal compared to the control, 0.5% (G2), and 2.5% (G6) groups, whereas the 1% charcoal group (G3) exhibited CAT activity comparable to other treatments.

The enhanced activity of key antioxidant enzymes suggests improved defense mechanisms against oxidative stress [38]. Enzymes such as GSH-Px, SOD, and CAT play critical roles in neutralizing reactive oxygen species, thus protecting cellular components from oxidative damage [39]. Specifically, these enzymes scavenge superoxide radicals (O_2_^•−^) and hydrogen peroxide (H_2_O_2_), preserving cellular membrane integrity *in vivo* [40].

Previous studies have corroborated these findings; Abdel-Tawwab et al. [9] reported that dietary charcoal improved fish health through radical scavenging activity. Ju et al. [41] observed increased SOD and myeloperoxidase activities in African catfish fed charcoal-supplemented diets. Similarly, enhanced GSH-Px activity was noted in Nile tilapia receiving charcoal at concentrations of 10–20 gm/kg feed [9], and African catfish showed significantly elevated antioxidant enzyme activities when fed charcoal-containing diets [41]. Wang et al. [6] suggested that the adsorption of toxic feed components by activated charcoal contributes indirectly to improved antioxidant status. Conversely, Zhang et al. [42] reported that supplementation with 450 mg/kg activated charcoal did not significantly affect radical scavenging activities (DPPH, ABTS^•+^, O_2_^•−^) in broiler chicks.

### Immunoglobulins

Serum immunoglobulin concentrations in mule ducks fed varying dietary charcoal levels are presented in Table 4. No significant differences were observed among all treatment groups for IgG and IgM concentrations (*p* > 0.05). However, ducks supplemented with 1.5% (G4) and 2% (G5) charcoal exhibited significantly elevated serum IgA levels (*p* = 0.0125) compared to the control group. The remaining charcoal-treated groups (0.5%, 1%, and 2.5%) showed intermediate IgA values without statistically significant differences relative to other groups. It is well-established that IgM constitutes the primary antibody generated upon initial antigen exposure, whereas secondary exposure elicits a robust IgG response. IgA, in contrast, predominates in mucosal secretions such as those of the intestinal, respiratory, and reproductive tracts, playing a critical role in mucosal immunity [37,43].

The enhancement of immune function following charcoal supplementation may be attributed to its capacity to adsorb harmful substances, including toxins, gases, and bacteria [4,5]. Activated charcoal’s binding affinity for mycotoxins and bacterial toxins can improve intestinal morphology, alleviate diarrhea, and reduce intestinal inflammation, thereby modulating systemic immune responses [42]. Bhatti et al. [44] also reported that charcoal mitigates mycotoxin-induced immunosuppression. Our findings align with Wang et al. [8], who observed increased serum and mucosal IgA levels in broilers supplemented with charcoal, whereas IgG levels remained unaffected. Similarly, Chu et al. [37] noted comparable effects on serum IgM in fattening pigs. Conversely, Ju et al. [41] documented significant elevations in serum IgM levels in catfish fed charcoal-supplemented diets. Zhang et al. [42], however, reported no significant effects of 450 mg/kg activated charcoal on broiler serum immunoglobulins.

### Lymphoid organs

The relative weights of lymphoid organs following dietary charcoal administration are summarized in Table 4. Supplementation with 1.5% and 2% charcoal significantly increased the relative spleen weight (*p* = 0.021), with the highest values observed in the 1.5% charcoal group and the lowest in the 0.5% group. No significant differences were detected in thymus and bursa of Fabricius weights across treatments (*p* = 0.879 and *p* = 0.986, respectively), although mean values showed a non-significant decrease in thymus and an increase in bursa weights in supplemented groups compared to controls.

Despite the absence of well-developed lymph nodes in most avian species, the spleen remains a vital lymphoid organ responsible for mounting immune responses [45]. These findings corroborate the immunoglobulin data presented herein, suggesting that charcoal-mediated toxin adsorption supports gut health and attenuates inflammation, thereby enhancing immune competence [42]. Comparable results have been reported by Jiya et al. [46], who observed similar trends in broilers receiving 0.5%–2% dietary charcoal. Furthermore, Khadem et al. [47] found that charcoal supplementation in aflatoxin-contaminated diets reduced spleen weight but increased bursa weight in broilers.

**Table 4.** Effect of graded levels of charcoal on immunoglobulins and the relative weight of ducks.

*p*-value	SEM	Charcoal levels						Traits
		G6 2.5%	G5 2.0%	G4 1.5%	G3 1.0%	G2 0.5%	G1 0%	
Serum immunoglobulin								
0.9254	0.512	5.55	5.64	5.71	5.39	5.42	5.12	IgG (mg/dl)
0.8471	0.281	2.54	2.49	2.44	2.51	2.29	2.38	IgM (mg/dl)
0.0125	0.184	1.80^ab^	1.91^a^	1.92^a^	1.46^b^	1.78^ab^	1.42^b^	IgA (mg/dl)
Lymphoid organs								
0.0207	0.034	0.192^b^	0.259^a^	0.264^a^	0.216^ab^	0.188^b^	0.222^ab^	Spleen, %
0.8785	0.062	0.444	0.453	0.457	0.415	0.466	0.472	Thymus, %
0.986	0.046	0.199	0.208	0.198	0.214	0.192	0.186	Bursa, %

^a, b^ means within row, followed by different superscripts, are significantly different (*p* ≤ 0.05). IgG = immunoglobulin G; IgM = immunoglobulin M; IgA = immunoglobulin A.

**Table 5.** Effect of graded levels of charcoal on intestinal morphology of ducks.

*p*-value	SEM	Charcoal levels						Traits
		G6 2.5%	G5 2.0%	G4 1.5%	G3 1.0%	G2 0.5%	G1 0%	
Duodenum								
0.0254	89.4	1,540.1^a^	1,546.4^a^	1,560.6^a^	1,450.9^ab^	1,188.0^b^	1,211.8^b^	Villus height (mm)
0.0502	3.81	70.08^ab^	74.91^a^	75.31^a^	69.96^ab^	60.11^b^	61.25^b^	Crypt depth (mm)
0.7216	3.32	21.98	20.64	20.72	20.74	19.76	19.78	V:C ratio
Jejunum								
0.7052	42.5	595.1	624.3	598.6	582.2	558.1	562.6	Villus height (mm)
0.1251	3.25	49.9	53.5	50.5	48.7	49.6	46.5	Crypt depth (mm)
0.5624	1.84	11.93	11.67	11.85	11.95	11.25	12.10	V:C ratio
Ileum								
0.4151	42.5	604.4	589.5	585.1	572.4	548.9	551.5	Villus height (mm)
0.1020	4.11	48.2	46.4	43.2	47.1	45.2	46.5	Crypt depth (mm)
0.1260	2.11	12.54	12.70	13.54	12.15	12.14	11.86	V:C ratio
Cecum								
0.0256	8.42	87.3^a^	89.5^a^	84.9^a^	70.9^b^	68.2^b^	69.4^b^	Villus height (mm)
0.0498	2.11	18.5^b^	19.1^b^	21.9^ab^	23.2^a^	22.9^a^	22.9^a^	Crypt depth (mm)
0.0481	0.68	4.72^a^	4.69^a^	3.88^ab^	3.06^b^	2.98^b^	3.03^b^	V:C ratio

^a,b^ Means within row, followed by different superscripts, are significantly different (*p* ≤ 0.05). V:C ratio = villus height to crypt depth ratio.

### Intestine morphology

As presented in Table 5, dietary supplementation with charcoal significantly influenced the morphology of the duodenum and cecum in Mule ducks, whereas the jejunum and ileum remained unaffected (*p* > 0.05). Specifically, inclusion of charcoal at 1.5%, 2%, and 2.5% enhanced villus height in the duodenum (*p* = 0.025) and increased crypt depth (*p* = 0.0502) relative to the control group. Similarly, cecal villus height was significantly elevated (*p* = 0.026) in ducks receiving 1.5%, 2%, and 2.5% charcoal compared to other treatment groups, including controls. Dietary charcoal at 2% and 2.5% (G5 and G6) reduced crypt depth (*p* = 0.0498) and increased the villus-to-crypt (V:C) ratio (*p* = 0.0481) compared to the control, 0.5%, and 1% charcoal groups.

The observed increases in villus height correspond to an expanded absorptive surface area, potentially improving nutrient uptake [48]. This improvement in duodenal and cecal morphology may result from charcoal’s capacity to adsorb intestinal toxins, thereby preventing their absorption and mitigating mucosal damage [3]. Consistent with these findings, Zhang et al. [42] demonstrated that charcoal’s adsorptive properties toward mycotoxins and bacterial toxins contribute to enhanced intestinal morphology, reduced diarrhea incidence, and alleviated intestinal inflammation. Similarly, Samanya and Yamauchi [49], as well as Yamauchi et al. [10], reported significant increases in intestinal villus height and epithelial cell area in White Leghorn hens supplemented with charcoal.

Conversely, Rattanawut et al. [50] and Hayajneh et al. [51] observed no significant effects of dietary bamboo charcoal (0.5% and 1.0%) on villus height or surface area in the small intestine of laying hens, indicating possible species-specific or dose-dependent responses.

## Conclusion

This study demonstrates that dietary supplementation with 1.5% activated charcoal confers multiple health benefits in ducks without eliciting adverse effects. Specifically, this inclusion level effectively supports renal function, enhances immune competence, augments antioxidant defense mechanisms, and promotes favorable modifications in intestinal morphology. These findings highlight the potential of activated charcoal as a safe and efficacious feed additive to improve overall physiological status and productivity in ducks. Conversely, supplementation at higher levels (2.5%) appeared to attenuate these positive outcomes, suggesting that excessive charcoal inclusion may be counterproductive. Future research should aim to elucidate the optimal dosage range and underlying mechanisms to maximize the benefits of charcoal supplementation in poultry nutrition.

## List of abbreviations

ALT, alanine transaminase; AST, alanine aminotransferase; CP, crude protein; IgA, immunoglobulin A; IgM, immunoglobulin M; MDA, malondialdehyde; ME, metabolizable energy; RBC, red blood cell; V:C, villus to crypt ratio; WBC, white blood cell.

## References

[1] Zhang Y, Lin Z, Wang L, Guo X, Hao Z, Li Z (2022). Cooperative interaction of phenolic acids and flavonoids contained in activated charcoal with herb extracts, involving cholesterol, bile acid, and Fxr/Pxr activation in broilers fed with mycotoxin-containing diets. Antioxidants.

[2] Zhao RS, Yuan JP, Jiang T, Shi JB, Cheng CG (2008). Application of bamboo charcoal as solid-phase extraction adsorbent for the determination of atrazine and simazine in environmental water samples by high-performance liquid chromatography-ultraviolet detector. Talanta.

[3] Anjaneyulu Y, Rao PR, Naidu NRG (1993). Experimental aflatoxicosis and its amelioration by activated charcoal in broiler chicken study on performance and haematology. J Vet Anim Sci.

[4] Kutlu HR, Ünsal I, Görgülü M (2001). Effects of providing dietary wood (Oak) charcoal to broiler chicks and laying hens. Anim Feed Sci Technol.

[5] Watarai S, Tana (2005). Eliminating the carriage of *Salmonella enterica* serovar enteritidis in domestic fowls by feeding activated charcoal from bark containing wood vinegar liquid (Nekka-Rich). Poult Sci.

[6] Wang L, Zhu L, Gong L, Zhang X, Wang Y, Liao J (2019). Effects of activated charcoal-herb extractum complex on antioxidant status, lipid metabolites and safety of excess supplementation in weaned piglets. Animals.

[7] Kim S-H, Lee I-C, Kang SS, Moon CJ, Kim SH, Shin DH (2011). Effects of bamboo charcoal and bamboo leaf supplementation on performance and meat quality in chickens. J Life Sci.

[8] Wang L, Zhang Y, Guo X, Gong L, Dong B (2022). Beneficial alteration in growth performance, immune status, and intestinal microbiota by supplementation of activated charcoal-herb extractum complex in broilers. Front Microbiol.

[9] Abdel-Tawwab M, El-Sayed GO, Shady SH (2017). Effect of dietary active charcoal supplementation on growth performance, biochemical and antioxidant responses, and resistance of Nile tilapia, *Oreochromis niloticus* (L.) to environmental heavy metals exposure. Aquaculture.

[10] Yamauchi K, Ruttanavut J, Takenoyama S (2010). Effects of dietary bamboo charcoal powder including vinegar liquid on chicken performance and histological alterations of intestine. J Anim Feed Sci.

[11] Farghly MF, Elsagheer AMA, Jghef MM, Taha AE, Abd El-Hack ME, Jaremko M (2023). Consequences of supplementing duck’s diet with charcoal on carcass criteria, meat quality, nutritional composition, and bacterial load. Poult Sci.

[12] Keithi-Reddy SR, Patel TV, Armstrong AW, Singh AK (2007). Uremic pruritus. Kidney Int.

[13] NRC (1994). Nutrient requirements of poultry.

[14] Heredia VIJ, Pérez ME, Salva AG, Robles CI, Hernández MB, Halloy M (2024). Hematology of *Liolaemus pacha* (Iguania: Liolaemidae) and its relationship with mite infestation, reproductive period and body condition. An Acad Bras Ciênc.

[15] Hartmann AE (1997). Tietz fundamentals of clinical chemistry. Arch Pathol Lab Med.

[16] Pandey M, Shah SV, Trivedi MM, Pathan MM, Lunagariya PM, Patel YG (2022). Effect of feeding rice distillers dried grains and mixture of non-legume and legume straw on hemato-biochemical and mineral profile of growing dairy heifers. Indian J Anim Nutr.

[17] Miranda MP (2024). Comparison of the effect of sodium chloride concentration on protein determination: Bradford and Biuret methods. Anal Biochem.

[18] Tanaka M, Tanaka S, Suzuki E, Kobayashi R, Takahashi S (2023). Effect of albumin measurement methods on the albumin–bilirubin grade. Ann Clin Biochem.

[19] Ayman AS, Abdou SG, Sadek EH, El-Kassim MAA (2024). Effect of date palm pollen as growth promoters on the performance of Ossimi lambs. Arch Agri Sci J.

[20] Resmi PE, Aarathi P, Suneesh PV, Ramachandran T, Bipin GN, Satheesh BT (2024). Development of a µPAD for the point-of-care testing of serum glutamic oxaloacetic transaminase (SGOT). Microchim Acta.

[21] Aboshama M, Abdo W, Elsawak A, Khater A (2024). Effect of the long-term use of a NOAEL dose of acetaminophen (paracetamol) on hepatic, renal, and neural tissues of aged albino rats. Open Vet J.

[22] Evans MG, Ribeiro GO, Henry DH, Johnson JA, Campbell JR, Waldner C (2023). Effect of sodium sulfate concentration in drinking water for beef heifers, and the *in vitro* effect of bismuth subsalicylate on H_2_S production and fiber disappearance. CAN J ANIM SCI.

[23] Yadav R, Yadav P, Singh G (2023). Uterine torsion in buffaloes-a complete review. Buf Bull.

[24] El-Sheshtawy SM, El-Zoghby AF, Shawky NA, Samak DH (2021). Aflatoxicosis in Pekin duckling and the effects of treatments with lycopene and silymarin. Vet World.

[25] Milevic A, Simic M, Tomovic M, Rankovic M, Jakovljevic V, Bradic J (2022). The effects of methanol extract of *Galium verum* L. on cardiac redox state in hypertensive rats. Braz J Pharm Sci.

[26] Ahmed AY, Aowda SA, Hadwan MH (2021). A validated method to assess glutathione peroxidase enzyme activity. Chem Pap.

[27] Gharib FAEL, Osama K, Sattar AMAE, Ahmed EZ (2024). Impact of *Chlorella vulgaris*
*Nannochloropsis salina*
*Arthrospira platensis* as bio-stimulants on common bean plant growth, yield and antioxidant capacity. Sci Rep.

[28] Almulaiky YQ (2025). Exploring the bioactive compounds in Capparis spinosa: antioxidant activities and enzymatic assays. Discov Plants.

[29] Mahrose KM, Abol-Ela S, Amin RM, Abou-Kassem DE (2022). Restricted feeding could enhance feed conversion ratio and egg quality of laying Japanese quail kept under different stocking densities. Anim Biotechnol.

[30] suvarna k s, layton c, bancroft j d (2013). Bancroft’s theory and practice of histological techniques.

[31] Tufarelli V, Losacco C, Tedone L, Passantino L, Tarricone S, Laudadio V (2023). Hemp seed (*Cannabis sativa* L.) cake as sustainable dietary additive in slow-growing broilers: effects on performance, meat quality, oxidative stability and gut health. Vet Q.

[32] Duncan CB (1955). Multiple range and multiple F-tests. Biometrics.

[33] SAS (2009). SAS user's guide, statistics. 9.2th edition. SAS Institute Inc.

[34] El-Kafoury B, Saleh NK, Saad DA, Shawky MK, Mehanna N, Ghonamy E (2019). Role of activated charcoal in limiting the progression of chronic kidney disease in albino rats. Al-Azhar Med J.

[35] Ruben NT, Raphaël KJ, Boris NT, Doriane YMD, Hervé MK, Serges K (2017). Performances of broiler chickens fed on diet supplemented with thyme and oregano essential oils stabilized in a plant charcoal matrix. J World Poult Res.

[36] Enyenihi GE, Ogbuzuru EN, Ojukwu ME (2022). Effects of supplementing broiler feed with coconut shell charcoal on their serum biochemical and haematological parameters, total coliform and bacterial counts. Niger J Anim Sci.

[37] Chu GM, Jung CK, Kim HY, Ha JH, Kim JH, Jung MS (2013). Effects of bamboo charcoal and bamboo vinegar as antibiotic alternatives on growth performance, immune responses and fecal microflora population in fattening pigs. Anim Sci J.

[38] Ballesteros ML, Wunderlin DA, Bistoni MA (2009). Oxidative stress responses in different organs of *Jenynsia multidentata* exposed to endosulfan. Ecol Toxicol Environ Saf.

[39] Kudlackova MK, Ursinyova M, Blazicek P, Spustova V, Ginter E, Hladikova V (2003). Free radical disease prevention and nutrition. Bratisl Lek Listy.

[40] Bounous GU, Sukkar S, Molson JH (2003). The antioxidant system. Anticancer Res.

[41] Ju K, Kil M, Ri S, Kim T, Zhang L, Yan M (2022). Dietary intake of bamboo vinegar and charcoal powder (BVC) enhances resistance of African catfish *Clarias gariepinus* to bacterial pathogen. J Oceanol Limnol.

[42] Zhang Y, Fu X, Wang L, Guo X, Dong B (2024). Sorption of phenols and flavonoids on activated charcoal improves protein metabolism, antioxidant status, immunity, and intestinal morphology in broilers. Front Vet Sci.

[43] McGlone J, Pond WG (2003). Pig production: biological principles and applications.

[44] Bhatti SA, Khan MZ, Saleemi MK, Hassan ZU (2021). Combating immunotoxicity of aflatoxin B1 by dietary carbon supplementation in broiler chickens. Environ Sci Pollut Res.

[45] Mast J, Goddeeris BM (1999). Development of immunocompetence of broiler chickens. Vet Immunol Immunopathol.

[46] Jiya EZ, Ayanwale BA, Adeoye AB, Kolo PS, Tsado DN, Alabi OJ (2014). Carcass yield, organoleptic and serum biochemistry of broiler chickens fed activated charcoal. J Agric Crop Res.

[47] Khadem AA, Sharifi SD, Barati M, Borji M (2012). Evaluation of the effectiveness of yeast, zeolite and active charcoal as aflatoxin absorbents in broiler diets. Glob Vet.

[48] Onderci M, Sahin N, Sahin K, Cikim G, Aydín A, Ozercan I (2006). Efficacy of supplementation of α-amylase-producing bacterial culture on the performance, nutrient use, and gut morphology of broiler chickens fed a corn-based diet. Poult Sci.

[49] Samanya M, Yamauchi K (2001). Morphological changes of the intestinal villi in chickens fed the dietary charcoal powder including wood vinegar compounds. J Poult Sci.

[50] Rattanawut J, Todsadee A, Rattanapan W (2021). Supplemental effect of bamboo charcoal and bamboo vinegar, alone or in combination, on laying hen performance, egg quality, intestinal bacterial populations and alteration of intestinal villi. Ital J Anim Sci.

[51] Hayajneh FMF, Abdelqader A, Zakaria H, Abuajamieh M, Araj SA (2024). Drug resistance and coccidiosis affects immunity, performance, blood micronutrients, and intestinal integrity in broiler chickens. Int J Vet Sci.

